# Supplementary material for: Differentiation of primate primordial germ cell-like cells following transplantation into the adult gonadal niche
Source: Nat Commun. 2018 Dec 17;9:5339. doi: 10.1038/s41467-018-07740-7 (PMC6297357; doi:10.1038/s41467-018-07740-7)
Supplement: Supplementary file 1 — Supplementary Information [file 41467_2018_7740_MOESM1_ESM.pdf]

Sosa *et al*. Differentiation of primate primordial germ cell-like cells following transplantation into the adult gonadal niche

Supplementary Information: 6 figures and 1 table

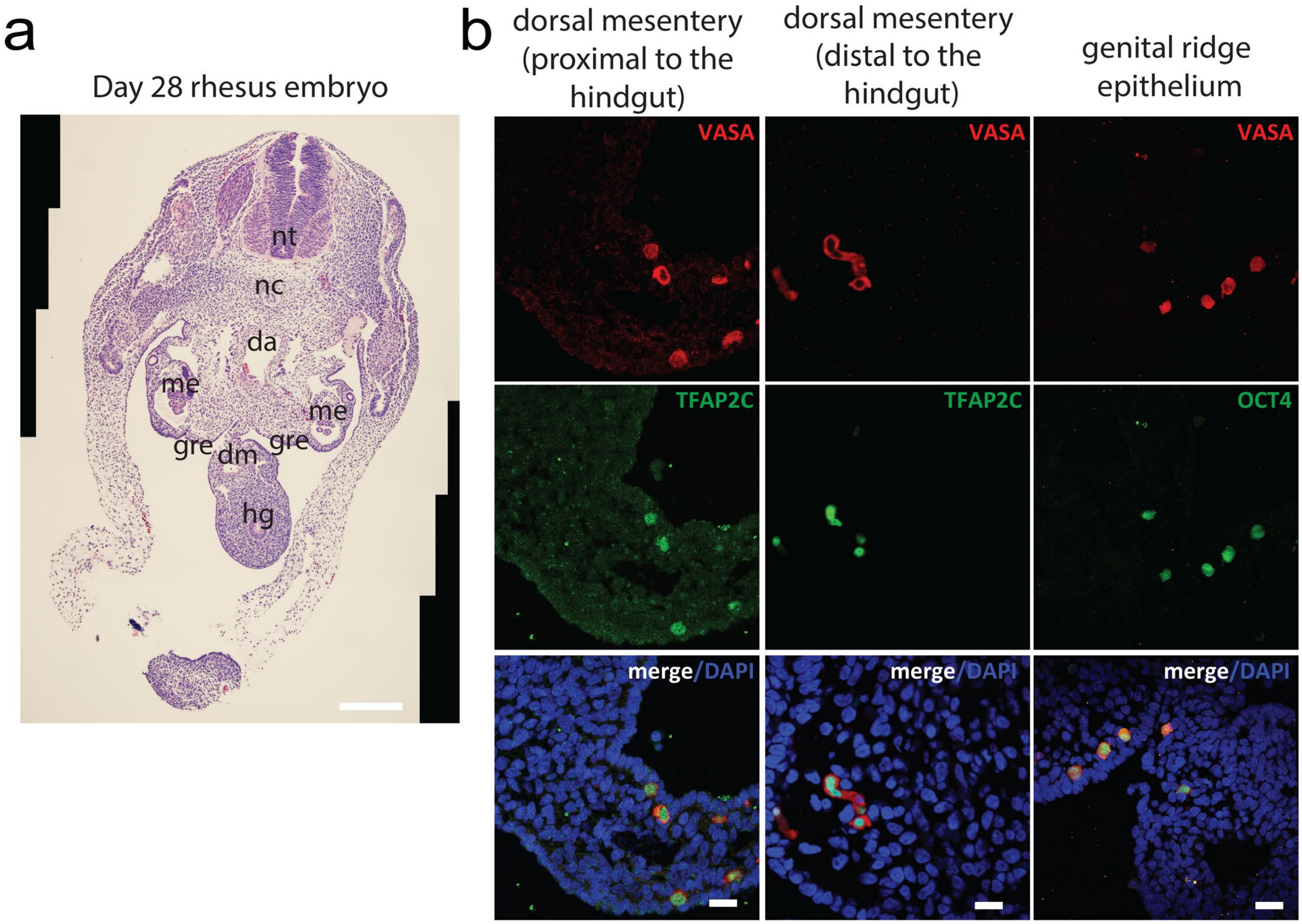

Supplementary Figure 1| rPGCs at CS12 co-express early and late rPGC markers. (a) H&E of a D28 embryo with key anatomical structures labeled; me=mesenephros, da=dorsal aorta, nc=notochord, nt=neural tube, gre=genital ridge epithelium, dm=dorsal mesentery; hg=hindgut. scale bars, 200µm. (b) The majority of TFAP2C (green) and OCT4 (green) positive D28 rPGCs co-express the late stage rPGC marker, VASA (red) while located in the dorsal mesentery and in the genital ridge epithelium. scale bars, 15µm. N=3 biological replicates of CS12, D28 embryos.

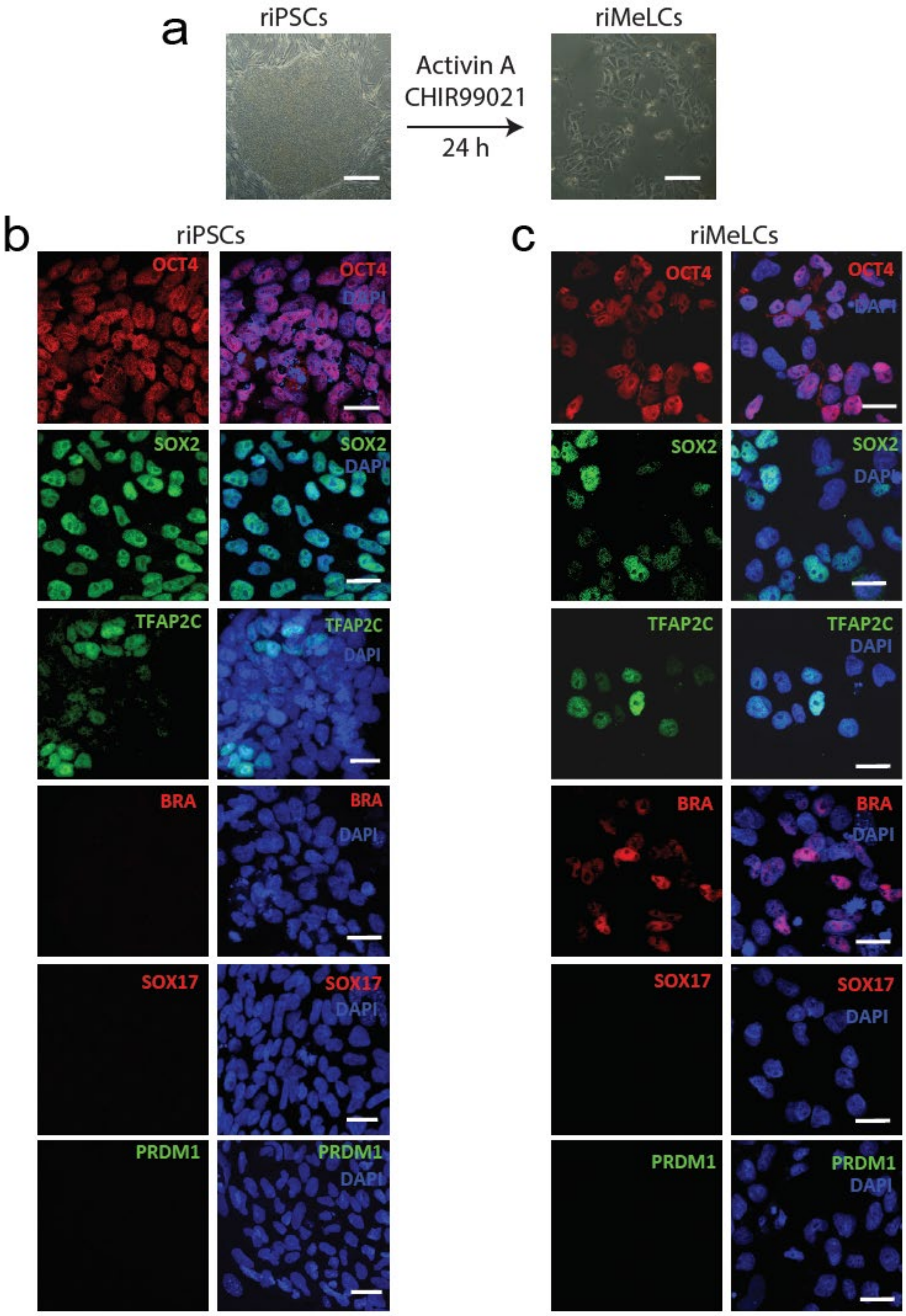

Supplementary Figure 2| SOX17 and PRDM1 not expressed in riPSCs or riMeLCs. (a) Induction of riMeLCS after 24 hours in media containing Activin A and CHIR99021. (b) IF staining of riPSCs colonies reveals homogenous expression of OCT4 (red) and SOX2 (green). In contrast, TFAP2C (green) is heterogeneously expressed. Brachyury (BRA), SOX17, and PRDM1 are undetectable in undifferentiated riPSCs. Scale bars,

20μm. (c) IF staining of riMeLCs reveals that unlike riPSCs, BRA (red) is induced and SOX2 (green) is down-regulated. SOX17, and PRDM1 protein were not detected in riMeLCs. Scale bars, 20μm. Shown are results using the riPSC89 line. N= 3 biological replicates of riPSC89 line.

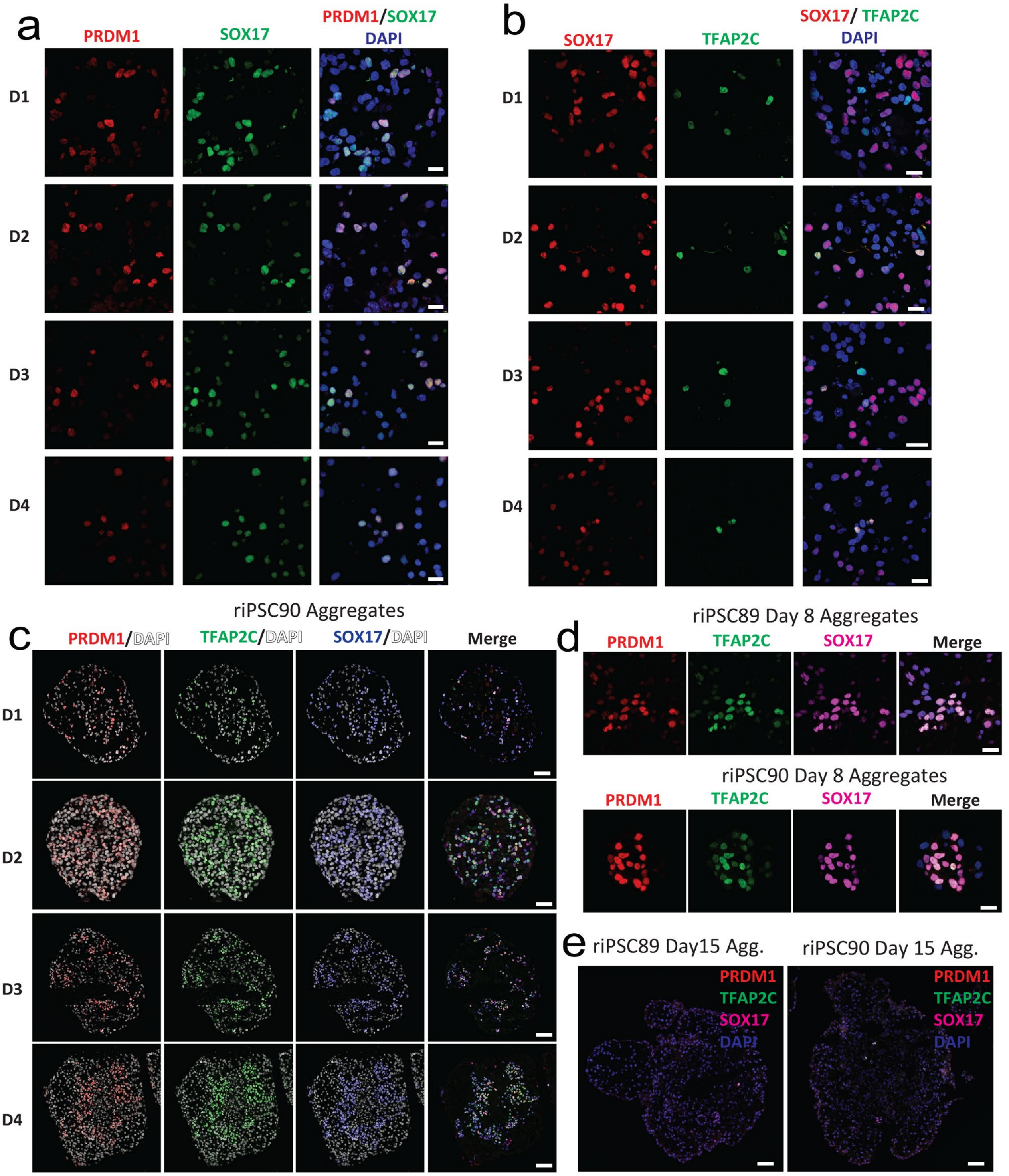

Supplementary Figure 3| PRDM1, TFAP2C, and SOX17 detected in rPGCLCs. (a) Double Immunofluorescence (IF) staining for PRDM1 (red) and SOX17 (green) and the nuclear stain DAPI (blue) identifies double positive cells at day (D) 1 of riPSC89

aggregate differentiation. Scale bars, 20µm. (b) Double IF staining for SOX17 (red) and TFAP2C (green) and the nuclear stain DAPI (blue), identifies double positive cells emerging between D1 and D2 of riPSC89 aggregate differentiation. Scale bars, 20µm. (c) Triple IF staining for PRDM1 (red), TFAP2C (green), SOX17 (blue) and the nuclear stain DAPI (white) identifies triple positive cells as early as D1 of aggregate differentiation in riPSC90. Scale bars, 40µm. (d) Triple IF staining of D8 aggregates for PRDM1 (red), TFAP2C (green), SOX17 (magenta) and the nuclear stain DAPI (blue) identified triple positive rPGCLCs from riPSC89 and riPSC90 lines. Scale bars, 20µm. (e) At day 15 of aggregate formation, no triple-positive rPGCLCs are identified in either riPSC89 or riPSC90. Scale bars, 40µm. N= 3 biological replicates of each riPSCs line (riPSC89 and riPSC90).

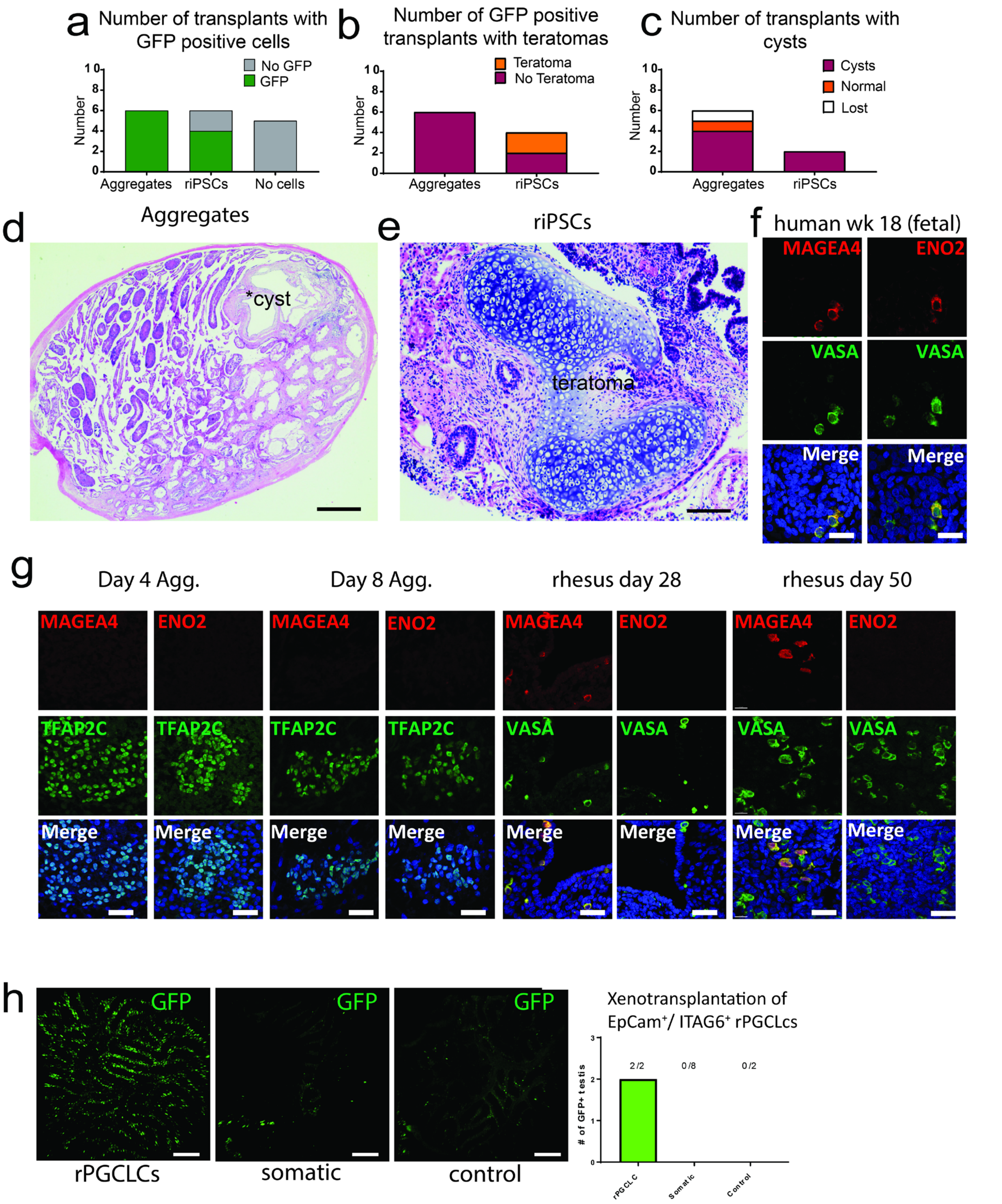

Supplementary Figure 4| Xenotransplantation of donor cells, riPSC89$^{UbiC:GFP}$. (a) Number of xenotransplants containing GFP positive ($^{+}$) cells. (b) Number of GFP^{+} xenotransplants with teratomas. (c) Number of GFP^{+} xenotransplants that formed cysts. (d) Histological section (H&E) of recipient mouse testicle which received aggregate cells

formed large cysts in 4/6 replicates (asterisk) near normal seminiferous tubules. (e) Histological section (H&E) of recipient mouse testicles that received riPSCs resulted in teratoma formation in 2/4 replicates. (f) Both MAGEA4 (red) and ENO2 (red) expression are observed in 18-week fetal hPGCs positive for VASA (green), and the nuclear marker DAPI (blue). (g) In contrast MAGEA4 (red) and ENO2 (red) expression are not observed in day (D) D4 or D8 aggregates containing TFAP2C^{+}(green) rPGCLCs. MAGEA4 (red) is detectable in D28 (CS12) and Day 50 (CS23) VASA positive rPGCs (green) and the nuclear marker DAPI (blue). Scale bars, 20μm. N= 3 biological replicates for D4, D8, and D28 (CS12); n=2 biological replicates for Day 50 (CS23) (h). Detection and quantification of testicles with GFP signal (green; donor cells) within recipient seminiferous tubules, following xenotransplantation with FACS isolated D4 rPGCLCs (EPCAM^{+}/ITGA6^{+}; n=2 testicles) or the aggregate somatic cells (EPCAM/ITGA6 double negative; n=8 testicles). The control group (sham; n=2 testicles) was not manipulated. Scale bars, 40μm.

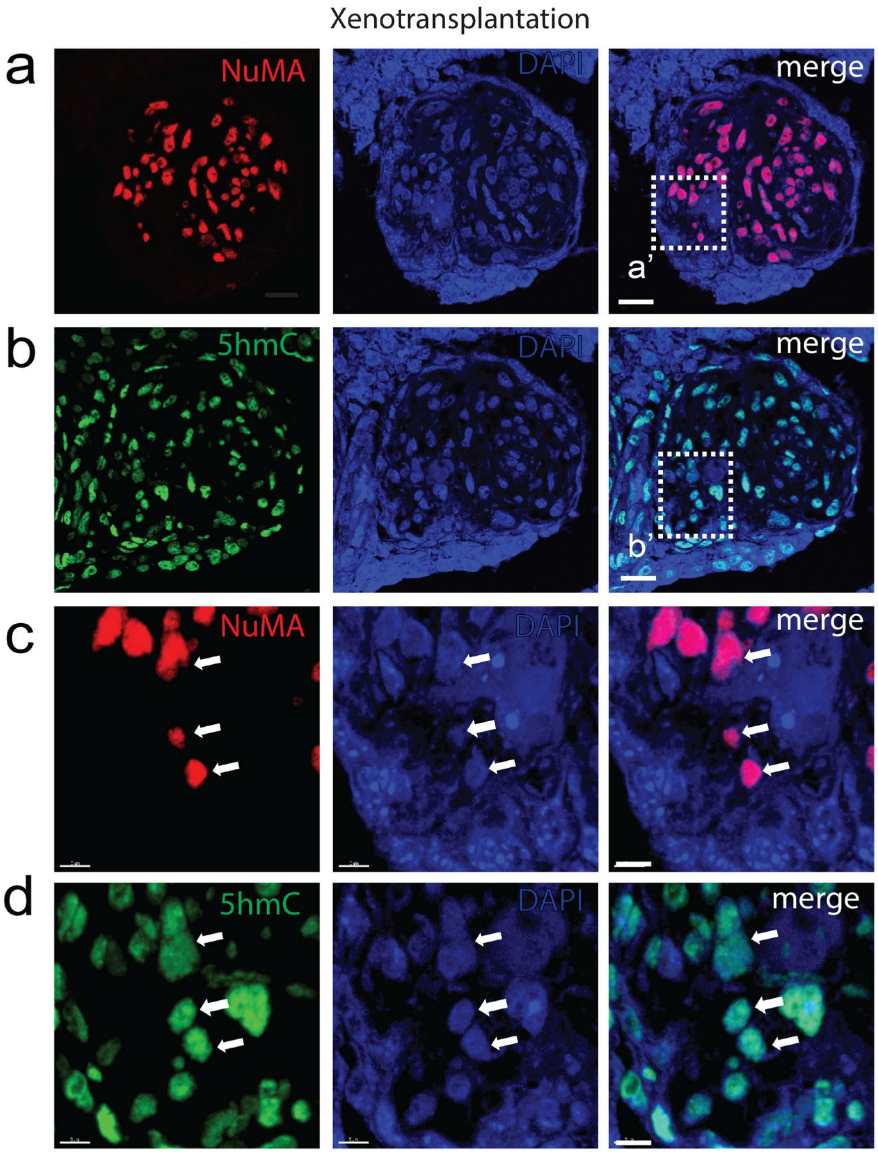

Supplementary Figure 5| Serial-section immunofluorescence on recipient testicles. (a) Detection of donor specific, NuMA- positive ($^+$) cells (red) in the seminiferous tubules of recipients.(b) 5hmC$^+$ cells (green) detected in the seminiferous tubules of recipients, in the corresponding serial section. Nuclei were detected using DAPI (blue). (c) Higher power view of cells within the white box of a', which detect NuMA (red). (d) Higher power view of cells with white box of b', which detect 5hmC (green). (c,d) Arrows identify donor cells (NuMA$^+$/ DAPI$^+$) detected in both serial sections, and therefore correspond to NuMA$^+$ cells that are enriched in 5hmC. Scale bars, 10μm. N= 13 transplanted recipient testicles.

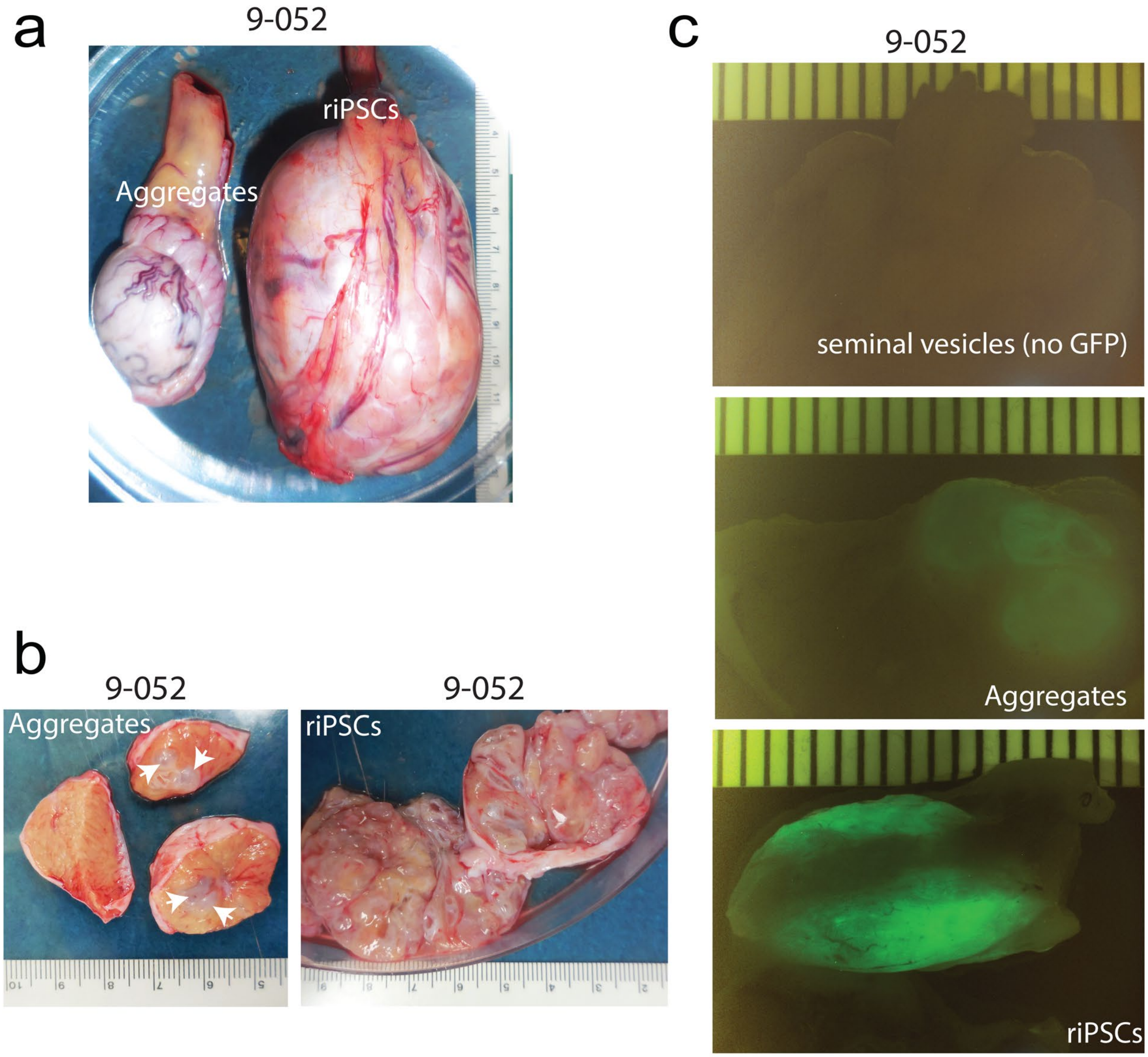

Supplementary Figure 6| Recipient testicles after homologous transplantation. (a) The size of the recipient testicle (animal 9-052) that received undifferentiated riPSCs was visually larger than the contralateral testicle that received donor aggregate cells. (b) Dissected testicle from animal 9-052, reveal the existence of cysts when aggregate cells were transplanted or teratoma when undifferentiated riPSCs were transplanted. c) Control seminiferous vesicles did not have GFP signal, whereas GFP was detected in the testicle that received aggregates and the testicle that received riPSCs.

Supplementary Table 1. List of antibodies used for experiments

Antibody	Company/Supplier	Catalog #	Dilution
mouse anti-5mC	Aviva Biosciences	AMM99021	1:100
goat anti-Oct4	Santa Cruz Biotechnology	sc8628, X	1:100
mouse anti-OCT3/4 (IgG2b)	Santa Cruz Biotechnology	sc5279	1:100
mouse anti-TFAP2C	Santa Cruz Biotechnology	sc12762	1:200
rabbit anti-TFAP2C	Santa Cruz Biotechnology	sc8977	1:200
rabbit anti-PRDM1	Cell Signaling Technology	9115S	1:100
goat anti-SOX17	Neuromics	GT15094	1:100
mouse anti-SOX2 (IgG2a)	R&D Systems	MAB2018	1:100
goat anti-VASA	R&D Systems	AF2030	1:100
goat anti-Brachyury/T	R&D Systems	AF2085	1:100
rabbit anti-Non Human Primate (NHP) cell	Dr. Orwig	N/A	1:200
mouse anti-MAGE A4	Dr. Spagnoli	Clone 57B	1:30
mouse anti-ENO2	BioLegend	MMS-518P	1:500
mouse anti-GFP	Cell Signaling Technology	2956S	1:100
rabbit anti-5hmC	Active Motif	39769	1:100
mouse anti-5hmC	Cell Signaling Technology	51660S	1:100
mouse anti-Ki67	Pharmingen	556003	1:200
rabbit-anti-NuMA	Abcam	Ab84680	1:200
AlexaFluor 488, Donkey anti-goat IgG (H + L)	JacksonImmunoResearch	705-546-147	1:200
AlexaFluor 488, Donkey anti-mouse IgG (H + L)	JacksonImmunoResearch	715-546-150	1:500
AlexaFluor 488, Donkey anti-rabbit IgG (H + L)	LifeTechnologies	A21206	1:500
AlexaFluor 488, Goat anti-mouse IgG2a	LifeTechnologies	A21131	1:200
AlexaFluor 594, Donkey anti-mouse IgG2b	LifeTechnologies	A21145	1:200
AlexaFluor 594, Donkey anti-goat IgG (H + L)	JacksonImmunoResearch	705-586-147	1:200
AlexaFluor 594, Donkey anti-rabbit IgG (H + L)	JacksonImmunoResearch	711-585-152	1:200
AlexaFluor 594, Donkey anti-mouse IgG (H + L)	LifeTechnologies	A21203	1:500
AlexaFluor 647, Donkey anti-goat IgG (H + L)	LifeTechnologies	A21447	1:200
AlexaFluor 647, Donkey anti-rabbit IgG (H + L)	LifeTechnologies	A31573	1:200
