# Supplementary material for: Differentiation of primate primordial germ cell-like cells following transplantation into the adult gonadal niche
Source: Nat Commun. 2018 Dec 17;9:5339. doi: 10.1038/s41467-018-07740-7 (PMC6297357; doi:10.1038/s41467-018-07740-7)
Supplement: Supplementary file 3 — Reporting Summary [file 41467_2018_7740_MOESM3_ESM.pdf]

nature research

Corresponding author(s): Amander T. Clark, ph:2133596399, email:clarka@ucla.edu

Reporting Summary

Nature Research wishes to improve the reproducibility of the work that we publish. This form provides structure for consistency and transparency in reporting. For further information on Nature Research policies, see Authors & Referees and the Editorial Policy Checklist.

Statistical parameters

When statistical analyses are reported, confirm that the following items are present in the relevant location (e.g. figure legend, table legend, main text, or Methods section).

n/a	Confirmed	
☐	☒	The exact sample size (*n*) for each experimental group/condition, given as a discrete number and unit of measurement
☐	☒	An indication of whether measurements were taken from distinct samples or whether the same sample was measured repeatedly
☐	☒	The statistical test(s) used AND whether they are one- or two-sided<br>*Only common tests should be described solely by name; describe more complex techniques in the Methods section.*
☒	☐	A description of all covariates tested
☒	☐	A description of any assumptions or corrections, such as tests of normality and adjustment for multiple comparisons
☐	☒	A full description of the statistics including central tendency (e.g. means) or other basic estimates (e.g. regression coefficient) AND variation (e.g. standard deviation) or associated estimates of uncertainty (e.g. confidence intervals)
☒	☐	For null hypothesis testing, the test statistic (e.g. *F*, *t*, *r*) with confidence intervals, effect sizes, degrees of freedom and *P* value noted<br>*Give P values as exact values whenever suitable.*
☒	☐	For Bayesian analysis, information on the choice of priors and Markov chain Monte Carlo settings
☒	☐	For hierarchical and complex designs, identification of the appropriate level for tests and full reporting of outcomes
☒	☐	Estimates of effect sizes (e.g. Cohen's *d*, Pearson's *r*), indicating how they were calculated
☐	☒	Clearly defined error bars<br>*State explicitly what error bars represent (e.g. SD, SE, CI)*

Our web collection on statistics for biologists may be useful.

Software and code

Policy information about availability of computer code

Data collection	Imaris 8.1(Bitplane) software was used to count the number of single, double, and triple positive cells in Figure 2. Imaris was also used to count the number of Ki67 positive cells from Figure 5. FACs data was collected using BD FACSDIVA Version 8.0.1. Image J version 1.51 was used to put together stitched images.
Data analysis	FlowJo (Treestar) was used to analyze all flow data collected on FACSDIVA. Prisim 7(Graph pad) was used to make all graphs.

For manuscripts utilizing custom algorithms or software that are central to the research but not yet described in published literature, software must be made available to editors/reviewers upon request. We strongly encourage code deposition in a community repository (e.g. GitHub). See the Nature Research guidelines for submitting code & software for further information.

Data

Policy information about availability of data

All manuscripts must include a data availability statement. This statement should provide the following information, where applicable:
- Accession codes, unique identifiers, or web links for publicly available datasets
- A list of figures that have associated raw data
- A description of any restrictions on data availability

All relevant data are available from the authors.

Field-specific reporting

Please select the best fit for your research. If you are not sure, read the appropriate sections before making your selection.

☒ Life sciences ☐ Behavioural & social sciences ☐ Ecological, evolutionary & environmental sciences

For a reference copy of the document with all sections, see nature.com/authors/policies/ReportingSummary-flat.pdf

Life sciences study design

All studies must disclose on these points even when the disclosure is negative.

Sample size	Sample size calculations were not performed. For key experiments in the publication at least 2 replicates were conducted, and the n is stated in each relevant figure legend.
Data exclusions	No data was excluded from analysis.
Replication	To verify reproducibility of our experimental findings we conducted at least 2 successful replications for each experiment. To further validate our findings we also performed critical experiments using two separate established rhesus iPSC lines (riPSC89 and riPSC90).
Randomization	Randomization was not performed given the complexity and length of the experiments carried out.
Blinding	Blinding was not performed given the complexity and length of the experiments carried out.

Reporting for specific materials, systems and methods

Materials & experimental systems

n/a	Involved in the study
☐	☒ Unique biological materials
☐	☒ Antibodies
☐	☒ Eukaryotic cell lines
☒	☐ Palaeontology
☐	☒ Animals and other organisms
☒	☐ Human research participants

Methods

n/a	Involved in the study
☒	☐ ChIP-seq
☐	☒ Flow cytometry
☒	☐ MRI-based neuroimaging

Unique biological materials

Policy information about availability of materials

Obtaining unique materials	Rhesus macque induced pluripotent stem cell lines, riPSC89 and riPSC90 were established at UCLA and are freely available with a signed Material Transfer Agreement.

Antibodies

Antibodies used	The following antibodies are commercially available and were used for Immunofluorescence : anti-5mC (Aviva Biosciences, AMM99021; 1:100), anti-5hmC (Active Motif, 39769; 1:100), anti-5hmC (Cell Signaling Tech, 51660S; 1:100), anti-OCT4 (Santa Cruz, sc8628x; 1:100), anti-OCT3/4 (Santa Cruz, sc5279; 1:100), anti-TFAP2C (Santa Cruz, sc12762; 1:200), anti-TFAP2C (Santa Cruz, sc8977; 1:200), anti-PRDM1 (Cell Signaling Tech, 9115S; 1:100), anti-SOX17 (Neuromics, GT15094; 1:100), anti-SOX2 (R&D Systems, MAB2018; 1:100), anti-VASA (AF2030; 1:100), anti-Brachyury/T (R&D Systems, AF2085; 1:200), anti-ENO2 (Biolegend, MMS-518P; 1:500), anti-Ki67 (Pharmingen, 556003; 1:200), anti-NuMA (Abcam, Ab84680; 1:200).The antibodies, anti-MAGEA4 (Clone 57B; 1:30) and anti-Non Human Primate (NHP) cell (1:200) are not commercially available but were provided to us upon request.
Validation	Commercially available antibodies have all been used extensively in other publications. The antibody MAGEA4 was provided to us by Dr. Spagnoli who has published extensively, using this antibody. The NHP antibody was provided to us by Dr. Orwig who has published using this antibody.

Eukaryotic cell lines

Policy information about cell lines

Cell line source(s)	The rhesus macaque induced pluripotent stem cell (riPSC) lines, riPSC89 and riPSC90 were generated from rhesus embryonic fibroblast (REF) lines REF89 and REF90, respectively. The riPSC89UbiC:GFP reporter line was generated through episomal delivery of the lentivirus GFP-IRES_PURO_cassette.
Authentication	riPSC89, riPSC89GFP, and riPSC90 were confirmed to have normal male (42XY) karyotypes. riPSC89, riPSC90, REF89, and REF90 lines have all been authenticated by short tandem repeat analysis.
Mycoplasma contamination	riPSC cell lines: riPSC89, riPSC89GFP, and riPSC90 were routinely tested for Mycoplasma contamination, logs are available upon request.
Commonly misidentified lines (See ICLAC register)	Commonly misidentified lines were not used.

Animals and other organisms

Policy information about studies involving animals; ARRIVE guidelines recommended for reporting animal research

Laboratory animals	Time-mated pregnant CF-1(Charles River) female mice were purchased from the vendor prior to being euthanized . Embryos were harvested to generate murine embryonic feeder cells(MEFs) to support iPSC cultures. MEFS were derived in compliances with the UCLA institutional animal care and use committee (IACUC) approval.<br>Mouse transplantation studies using irradiated nude mice were approved by The University of Texas M. D. Anderson Cancer Center IACUC.<br>All rhesus macaque transplantation studies were approved by The University of Texas M. D. Anderson Cancer Center IACUC.<br>Mouse transplantation studies performed on busulfan-treated nude mice were approved by the IACUC committees of Magee-Womens Research Institute and the University of Pittsburgh School of Medicine.<br>All rhesus macaque time-mated breeding experiments were conducted following approval of the Oregon National Primate Research Center IACUC.
Wild animals	The study did not include wild animals.
Field-collected samples	The study did not include wild animals captured in the field

Flow Cytometry

Plots

Confirm that:

- [x] The axis labels state the marker and fluorochrome used (e.g. CD4-FITC).
- [x] The axis scales are clearly visible. Include numbers along axes only for bottom left plot of group (a 'group' is an analysis of identical markers).
- [x] All plots are contour plots with outliers or pseudocolor plots.
- [x] A numerical value for number of cells or percentage (with statistics) is provided.

Methodology

Sample preparation	Experiments carried out on aggregates containing primordial germ cell like cells (PGCLCs) from Day (D) 1 to D4 were digested using 0.05% trypsin. Due to the nature of the aggregates at D6 and D8, these samples were digested with Collagenase IV and 0.25% Trypsin to facilitate the production of a single cell suspension.
Instrument	Cells were collected using an BD ARIA-H Fluorescence Activated Cell sorter (FACs)
Software	FlowJo (V10) was used to analyze the data collected on the ARIA-H FACs (BD FACSDIVA software)
Cell population abundance	Double positive cells for EPCAM and ITAG6 were collected for at least two replicates at each individual time point with the percent of double positive cells is listed in the right hand corner of each contour plot, for each respective time point.
Gating strategy	To discriminate cells (population(p) 1= all events) from debris we set the gating axises to SSC-A/FSC-A. Next to gate out doublets we used SSC-W/SSC-H (P2) and FSC-W/FSC-H (P3). 7AAD was added prior to sorting and was used to gate out live (P4) vs dead cells. Lastly P5 was calculated from P4 (live cells), and was based on expression of both EPCAM or ITAG6. All single color and FMO controls were used to setup the gating strategy used to sort PGCLCs.

- [ ] Tick this box to confirm that a figure exemplifying the gating strategy is provided in the Supplementary Information.
